# Effect of *KCNQ1* rs2237892 polymorphism on the predisposition to type 2 diabetes mellitus: An updated meta-analysis

**DOI:** 10.1186/s13098-021-00683-y

**Published:** 2021-07-08

**Authors:** Hong-Liang Jiang, Han Du, Ying-Jun Deng, Xue Liang

**Affiliations:** 1 Department of Anorectal Medicine, Gaozhou Hospital of Traditional Chinese Medicine, Guangzhou University of Chinese Medicine, Gaozhou, 525025 Guangdong China; 2 Dermatology Department of Gaozhou Hospital of Traditional Chinese Medicine, Guangzhou University of Chinese Medicine, No. 32 Maoming Avenue, Gaozhou, 525025 Guangdong China; 3 The First Affiliated Hospital of Guangzhou University of Chinese Medicine, Guangzhou, 510006 Guangdong China; 4 Department of Science and Education, Gaozhou Hospital of Traditional Chinese Medicine, Guangzhou University of Chinese Medicine, Gaozhou, 525025 Guangdong China

**Keywords:** *KCNQ1* rs2237892, Polymorphism, T2DM, Meta-analysis

## Abstract

**Objectives:**

Previous studies have analyzed the potential effect of *KCNQ1* rs2237892 polymorphism on the predisposition to type 2 diabetes mellitus, but the findings are inconclusive and the subject of debate. The purpose of our study was to provide further insight into the potential association between *KCNQ1* rs2237892 polymorphism and the risk of type 2 diabetes mellitus.

**Methods:**

In total, 50 articles (60 studies) with 77,276 cases and 76,054 controls were utilized in our analysis. The pooled odds ratio (OR), 95% confidence interval (95% CI), and *p* value were used to evaluate the significance of our findings. Funnel plots and Beggar’s regression tests were utilized to determine the presence of publication bias.

**Results:**

Our meta-analysis results indicated that *KCNQ1* rs2237892 polymorphism could be correlated with the risk of type 2 diabetes mellitus under the C allelic, recessive, and dominant genetic models (OR = 1.25, 95% 1.19–1.32, *p* < 0.001; OR = 1.50, 95% CI 1.34–1.68, *p* < 0.001; OR = 1.26, 95% CI 1.14–1.40, *p* < 0.001, respectively). Additionally, ethnicity analysis revealed that the source of control, case size, and Hardy–Weinberg Equilibrium status were correlated to the polymorphism in the three genetic models.

**Conclusions:**

Our meta-analysis demonstrated significant evidence to support the association between *KCNQ1* rs2237892 polymorphism and predisposition to type 2 diabetes mellitus.

## Background

The worldwide prevalence of type 2 diabetes mellitus (T2DM) is increasing, along with associated comorbidities such as cardiovascular disease [1]. The International Diabetes Federation (IDF) reports that there were 9.3% (463 million) adults with diabetes in 2019, and 700 million people will have diabetes by 2045 [2]. Researchers consider T2DM to be a polygenic metabolic disorder with genetic heterogeneity that is affected by nongenetic (environmental), genetic, and lifestyle factors. However, the pathogenesis of T2DM still remains unclear [3].

Previous studies have reported that the potassium voltage-gated channel KQT-like sub-family, member 1 gene (*KCNQ1*) is associated with T2DM in Japanese, Korean, Chinese, Indian, and European populations [4–7]. Case–control studies investigating the role of *KCNQ1* polymorphisms in T2DM, have indicated that rs2237892, a single nucleotide polymorphism (SNP) located on intron 15, has a strong association with T2DM. Therefore, rs2237892 has been widely investigated in subsequent studies. However, there are disagreements between the different studies, and their validity has been limited by insufficient sample size and lack of ethnic diversity in the study populations [8–11]. Although a previous meta-analysis in 2012 investigated the association between *KCNQ1* rs2237892 polymorphism and T2DM risk, the authors only utilized 25 articles [12]. Therefore, our objective in the present meta-analysis, was to further examine and elucidate the connection between *KCNQ1* rs2237892 polymorphism and an increased risk of T2DM.

## Methods

### Publication search

We systematically searched for relevant publications published through March 11, 2021 using Cochrane Library, PubMed, EMBASE, Web of Science, and China National Knowledge Infrastructure. We used the following search terms: (“*KCNQ1*”, OR “potassium voltage-gated channel”, OR “KQT-like subfamily, member 1”, OR “rs2237892”) AND (“variant”, OR “polymorphism”, OR “mutation”) AND (“T2DM”, OR “type 2 diabetes mellitus”, OR “type 2 diabetes”, OR “T2D”). Two investigators independently checked the references of retrieved articles to select the publications they would include in the analysis.

## Selection criteria

Studies were selected according to the following inclusion criteria: full text could be found; the case–control studies focused on the relevance of *KCNQ1* rs2237892 polymorphism and T2DM risk; the *KCNQ1* rs2237892 genotype was obtained, and association between the *KCNQ1* rs2237892 SNP and T2DM prevalence was assessed. Studies were excluded if they met the following exclusion criteria: they were repetitions of other articles; they were not case–control studies; they were unpublished studies, conference articles, meta-analyses, systematic evaluations, and they were pedigree studies. We consulted the Preferred Reporting Project (PRISMA) Guide for Systematic Evaluation and Meta-Analysis to comply with standards for conducting and presenting results from meta-analyses [13].

## Data extraction

Referring to the inclusion/exclusion criteria, two investigators independently extracted data that included: first author, country, publication year, amount of cases and controls, Hardy–Weinberg equilibrium (HWE), control group source, and the availability of *KCNQ1* rs2237892 genotype. Only articles with maximum sample size were selected when similar data appeared in multiple publications. A third investigator reviewed the final results to ensure data accuracy, and discussions were held to resolve any conflicts.

## Study quality assessment

Two investigators performed independent quality assessments for each eligible article according to the 9-point Newcastle–Ottawa Scale [14]. The third investigator resolved any conflicting results produced by the two investigators. The assessment score included these criteria: case and control selection (4 points); confounding factor quality corrected in cases and controls (2 points), exposure ascertainment (3 points). The total scores ranged from 0 to 9, and scores > 6 were indicative of high-quality articles.

## Statistical analysis

We estimated the significance of the data describing *KCNQ1* rs2237892 SNP and T2DM risk using the OR and 95% CI. The Chi-Square-Based Q-test and I-Squared test were utilized to analyze the heterogeneity with *p* < 0.1 suggesting heterogeneity [15, 16]. We estimated the pooled OR by fixed effect model (Mantel–Haenszel) when no heterogeneity existed, or by the DerSimonian and Laird random effects model [17, 18]. We performed the Chi-squared test in controls, to examine HWE. To estimate the influence of the pooled ORs caused by an individual data set, we performed sensitivity analysis for each of the comparison models. The publication bias was tested by Funnel plot and Begg linear regression (19, 20), and Stata 12.0 was used to perform all analyses.

## Results

### Study characteristics

**Fig. 1:**
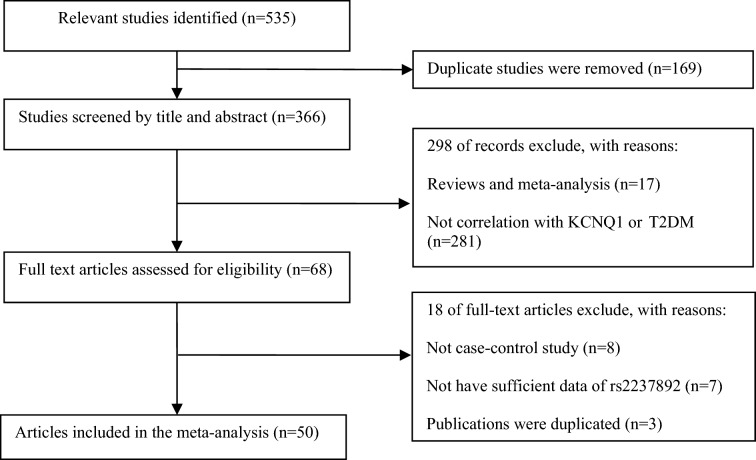
The flow sheet of identification of eligible studies

Figure 1 shows flowcharts of the selection of publications for the present study. There were 535 publications located in several electronic databases. After examining the research title, content, and abstract of the publications, the two investigators excluded 169 duplicate documents, 298 irrelevant papers, and examined the remaining 68 articles in full. Finally, our meta-analysis included 50 (60 case–control) publications. Among the 60 case–control studies, 51 included Asian populations, 4 included Caucasian, and 5 involved other populations. Of the studies in our meta-analysis, 24 were based on population (PB), 19 were based on hospital (HB), and 17 studies were based on no report (NR). The sample group of 21 studies was less than 500 patients, 10 studies included between 500 and 1000 patients, 4 studies included between 1001 and 2000 patients, and the remaining 6 studies had a sample group of greater than 2000. HWE balance (*p* < 0.05) was not met in 5 of the control groups. Due to lack of control group descriptions, 19 studies did not meet HWE assessment. Table 1 shows the main features of the study and the genotype distribution results of the HWE test.

**Table 1:** Studies and data included in this meta- analysis

Authors	Year	Country	Source of control	Sample size		Cse					Control					NOS score	HWE
				Case	Control	CC	CT	TT	C	T	CC	CT	TT	C	T		
Yasuda K et al	2008	Japanese	NR	2954	2988				5954	2802				5206	3618	7	NK
Yasuda K et al	2008	China/ Korea	NR	6552	6621				9042	4062				8078	5164	7	NK
Yasuda K et al	2008	European	NR	63	752				120	6				1399	105	7	NK
Lee YH et al	2008	Korea	HB	865	496	389	377	99	1155	575	182	239	75	603	389	7	0.811
Chen Z et al	2009	China	HB	57	341	27	24	6	78	36	162	144	35	468	214	6	0.720
Takeuchi F et al	2009	Japan	NR	519	503	228	236	55	692	346	183	244	76	610	396	7	0.717
Takeuchi F et al	2009	Japan	NR	1110	1014	492	488	130	1472	748	398	452	164	1248	780	7	0.063
Takeuchi F et al	2009	Japan	NR	3990	4878	1740	1785	465	5265	2715	1695	2345	838	5735	4021	7	0.580
Qi Q et al	2009	China	PB	424	1908				617	231				2576	1240	8	NK
Qi Q et al	2009	China	PB	152	1183				235	69				1614	752	8	NK
Hu C et al	2009	China	PB	1719	1720	947	643	129	2537	901	706	816	198	2228	1212	8	0.101
Liu Y et al	2009	China	NR	1880	1996	902	813	165	2617	1143	853	919	224	2625	1367	7	0.318
Tan JT et al	2009	Chinese	PB	1541	2196				2127	955				2943	1449	7	NK
Tan JT et al	2009	Malay	PB	1076	2257				1549	603				3070	1444	7	NK
Tan JT et al	2009	Indian	PB	246	364				482	10				684	44	7	NK
Zhang S et al	2009	China	HB	104	98	52	44	8	148	60	42	46	10	130	66	7	0.615
Yamauchi T et al	2010	Japanese	PB	4878	3345				6439	3317				4108	2582	7	NK
Yamauchi T et al	2010	Japanese	PB	2886	3087				3861	1911				3772	2402	7	NK
Han X et al	2010	China	PB	990	959	525	396	69	1446	534	415	437	107	1267	651	8	0.616
Xu M et al	2010	China	PB	1825	2200				2548	1102				2820	1580	8	NK
Zhou JB et al	2010	China	PB	537	510				773	301				663	357	8	NK
Been LF et al	2011	India	PB	1290	1019	1259	30	1	2548	32	982	36	1	2000	38	8	0.269
Been LF et al	2011	US-India	PB	139	557	133	6	0	272	6	523	32	2	1078	36	8	0.055
Saif-Ali R et al	2011	Malaysia	HB	234	177	135	79	20	349	119	81	75	21	237	117	7	0.572
Tabara Y et al	2011	Japan	NR	493	394	243	206	44	692	294	136	193	65	465	323	8	0.803
Saif-Ali R et al	2011	Malaysia	HB	300	230	183	99	18	465	135	113	90	27	316	144	7	0.171
Da W et al	2011	China	PB	223	201	115	92	16	322	124	79	88	34	246	156	8	0.268
Dai XP et al	2012	China	NR	367	212	233	112	22	578	156	110	82	20	302	122	7	0.412
Yu W et al	2012	China	PB	5409	614	2773	2245	391	7791	3027	235	313	66	783	445	8	0.011
Yu W et al	2012	China	PB	2994	3256	1608	1162	224	4378	1610	1391	1490	375	4272	2240	8	0.426
Gamboa-Melendez MA et al	2012	Mexico	HB	1027	990				1479	575				1313	667	7	NK
Turki A et al	2012	Tunisia	NR	883	591	763	106	14	1632	134	528	57	6	1113	69	7	0.003
Iwata M et al	2012	Japan	HB	724	763	342	300	82	984	464	283	329	151	895	631	8	0.002
Van Vliet-Ostaptchouk JV et al	2012	Netherlands	NR	4511	5152	4149	348	14	8646	376	4638	507	7	9783	521	7	0.073
Odgerel Z et al	2012	China	PB	177	216				223	131				315	117	8	NK
Gao X et al	2012	China	HB	200	200	95	88	17	278	122	72	102	26	246	154	8	0.276
Yamakawa-Kobayashi K et al	2012	Japan	PB	333	417				426	240				484	350	8	NK
Tam CH et al	2013	China	PB	5882	2569				8458	3306				3371	1767	7	NK
Almawi WY et al	2013	Lebanon	NR	994	1077	499	371	124	1369	619	801	225	51	1827	327	7	0.000
Long J et al	2013	America	PB	1551	2725				2823	279				4851	600	8	NK
Lin YD et al	2013	China	PB	2899	3261	1491	1174	234	4156	1642	1433	1431	397	4297	2225	7	0.174
Yang HL et al	2013	China	HB	222	140	123	87	12	333	111	60	59	21	179	101	8	0.308
Wang T et al	2013	China	HB	300	200	150	132	18	432	168	72	102	26	246	154	8	0.276
Bazzi MD et al	2014	Saudi	HB	78	96	71	7	0	149	7	89	7	0	185	7	7	0.711
The STDC	2014	Mexico/USA	NR	4366	3848				6435	2297				5487	2209	6	NK
Zhu AN et al	2014	China	HB	238	240	106	118	14	330	146	109	98	33	316	164	6	0.153
Zhang WL et al	2015	China	NR	530	452	274	217	39	765	295	194	192	66	580	324	8	0.104
Qian Y et al	2015	China	PB	2925	3281	1504	1185	236	4193	1657	1442	1440	399	4324	2238	8	0.177
Cui LJ et al	2016	China	HB	100	100	39	46	15	124	76	53	35	12	141	59	7	0.113
Zhou XY et al	2016	China	HB	305	200	148	136	21	432	178	72	102	26	246	154	7	0.276
Riobello C et al	2016	Spain	HB	180	501	155	25	0	335	25	450	51	0	951	51	6	0.230
Al-Shammari MS et al	2017	Saudi	NR	330	516	319	9	2	647	13	496	15	5	1007	25	7	0.000
Plengvidhya N et al	2018	Thailand	HB	500	500	285	192	23	762	238	254	205	41	713	287	8	0.968
Chen JF et a	2018	China	HB	84	104	34	42	8	110	58	57	36	11	150	58	7	0.155
Huang Q et al	2018	China	PB	506	497	250	220	36	720	292	215	231	51	661	333	8	0.336
Yang KL et al	2018	China	PB	522	522	270	215	37	755	289	237	232	53	706	338	8	0.732
Li YH et al	2018	China	NR	284	99	210	68	6	488	80	84	15	0	183	15	8	0.415
Li YH et al	2018	China	NR	293	208	144	128	21	416	170	88	97	23	273	143	8	0.628
Xu T et al	2018	China	HB	100	100	31	45	24	107	93	32	41	27	105	95	8	0.075
Totomoch-Serra A et al	2018	Mexico	HB	415	416				523	307				541	291	8	NK

## Meta-analysis results

The meta-analysis included 153,330 participants (77,276 cases and 76,054 controls). *KCNQ1* rs2237892 polymorphism was significantly associated with T2DM risk under the C allelic, recessive, and dominant genetic models (OR:1.25, 1.50 and 1.26; 95% CI 1.19–1.32, 1.34–1.68, and 1.14–1.40; *p* < 0.001, respectively). In ethnic subgroup analysis shown in Table 2, *KCNQ1* rs2237892 polymorphism was correlated with increased risk of T2DM in the dominant genetic model of East Asians, in the C allelic genetic model of East Asians, and in the C allelic genetic model of West Asian populations (OR = 1.39, 1.32 and 1.25; 95% CI 1.31–1.49, 1.27–1.37 and 1.19–1.32; *p* < 0.001, respectively). In the stratified analysis by source of control, marked correlation was found in the C allelic genetic model (HB, PB, and NR: OR = 1.24, 1.25 and 1.16; 95% CI 1.14–1.37, 1.19–1.32 and 1.02–1.32; *p* < 0.001, respectively) and the dominant genetic model (HB and PB: OR = 1.25 and 1.48; 95% CI 1.08–1.46 and 1.38–1.59, *p* < 0.05, respectively). In the case size stratification, the C allelic genetic model (OR = 1.23, 1.14, 1.25 and 1.33; 95% CI 1.09–1.38, 0.88–1.48, 1.19–1.32 and 1.27–1.39; *p* < 0.001, respectively), the dominant genetic model (OR = 1.24, 1.13, 1.41 and 1.43; 95% CI 1.05–1.46, 0.81–1.58 and 1.33–1.53; *p* < 0.001, respectively) and the recessive genetic model (500–1000: OR = 1.32, 95% CI 0.91–1.91, *p* < 0.001) found notable association between *KCNQ1* rs2237892 polymorphism and increased T2DM risk. Finally, we stratified by sample size—significant correlation was found in the C allelic genetic model (< 500, 1001–2000 and > 2000: OR = 1.23, 1.25 and 1.33; 95% CI 1.09–1.38, 1.19–1.32 and 1.27–1.39; *p* < 0.001, respectively) and the dominant genetic model (< 500, 1001–2000 and > 2000: OR = 1.24, 1.41 and 1.43; 95% CI 1.05–1.46, 1.14–1.75 and 1.33–1.53; *p* < 0.001, respectively).

**Table 2:** Pooled ORs and 95% CIs of the association between *KCNQ1* rs2237892 polymorphism and T2DM

Total and subgroups	Studies	CC vs CT + TT				CC + CT vs TT				C VS T			
		OR	95%CI	I^2^	P	OR	95%CI	I^2^	P	OR	95%CI	I^2^	P
Total	41/60	1.26	1.14 –1.40	87.2%	< 0.001	1.50	1.34–1.68	66.6%	< 0.001	1.25	1.19 –1.32	86.6%	< 0.001
Ethnicity													
East Asian	30/42	1.39	1.31–1.49	61.4%	< 0.001	1.59	1.50–1.68	0.0%	0.575	1.32	1.27–1.37	69.4%	< 0.001
Southeast Asian	3/4	1.43	1.20 –1.72	0.00%	0.453	1.79	1.27–2.52	0.0%	0.712	1.30	1.17–1.45	20.0%	0.290
South Asian	1/2	1,53	0.94–2.48	−	−	1.26	0.08–20.27	–	–	2.07	1.03 –4.17	64.6%	0.093
West Asian	3/3	0.64	0.26–1.57	82.3%	0.003	1.50	1.39–1.68	68.5%	0.075	1.25	1.19 –1.32	83.0%	0.003
Caucasian	2/4	1.00	0.56–1.76	79.0%	0.029	0.44	0.18–1.08	–	–	1.19	1.02–1.38	36.7%	0.192
Other	2/5	0.91	0.52–1.61	43.5%	0.184	0.68	0.27–1.70	0.0%	0.677	1.06	0.90–1.25	75.1%	0.003
Source of control													
HB	17/19	1.25	1.08–1.46	59.2%	0.001	1.68	1.44–1.97	10.6%	0.335	1.24	1.14–1.37	63.4%	< 0.001
PB	11/24	1.48	1.38–1.59	47.4%	0.040	1.50	1.34–1.68	0.0%	0.984	1.25	1.19–1.32	67.6%	< 0.001
NR	13/17	1.13	0.87–1.42	94.6%	< 0.001	1.21	0.91–1.62	86.2%	< 0.001	1.16	1.02–1.32	95.0%	< 0.001
Case size													
< 500	21	1.24	1.05–1.46	63.6%	< 0.001	1.77	1.50 –2.08	0.0%	0.483	1.23	1.09–1.38	71.5%	< 0.001
500 –1000	10	1.13	0.81–1.58	95.3%	< 0.001	1.32	0.91–1.91	88.5%	< 0.001	1.14	0.88–1.48	95.7%	< 0.001
1001–2000	4	1.41	1.14–1.75	82.4%	0.001	1.44	1.26–1.65	0.0%	0.670	1.25	1.19 –1.32	67.3%	< 0.001
> 2000	6	1.43	1.33–1.53	58.0%	0.036	1.56	1.41–1.72	35.2%	0.173	1.33	1.27–1.39	80.1%	< 0.001
HWE status													
Yes	36/36	1.36	1.28–1.45	57.3%	< 0.001	1.57	1.48 –1.67	2.9%	0.420	1.32	1.26–1.38	53.2%	< 0.001
No	5/5	0.95	0.46–1.96	97.8%	< 0.001	0.99	0.45 –2.18	94.0%	< 0.001	1.25	1.19–1.32	98.1%	< 0.001
NK	0/19	–	–	–	–	–	–	–	–	1.25	1.18 –1.34	82.8%	< 0.001

## Discussion

**Fig. 2:**
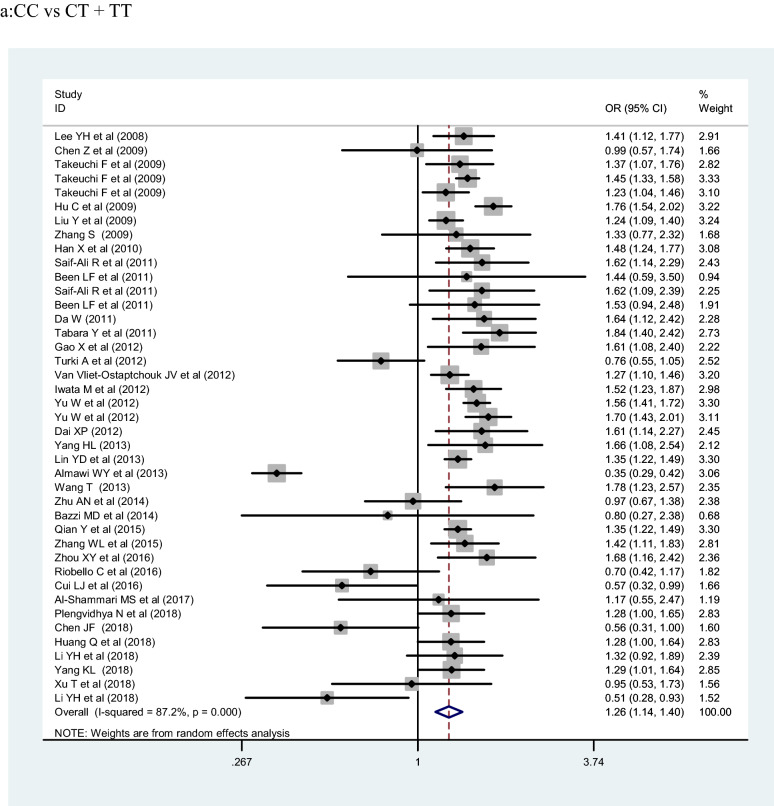

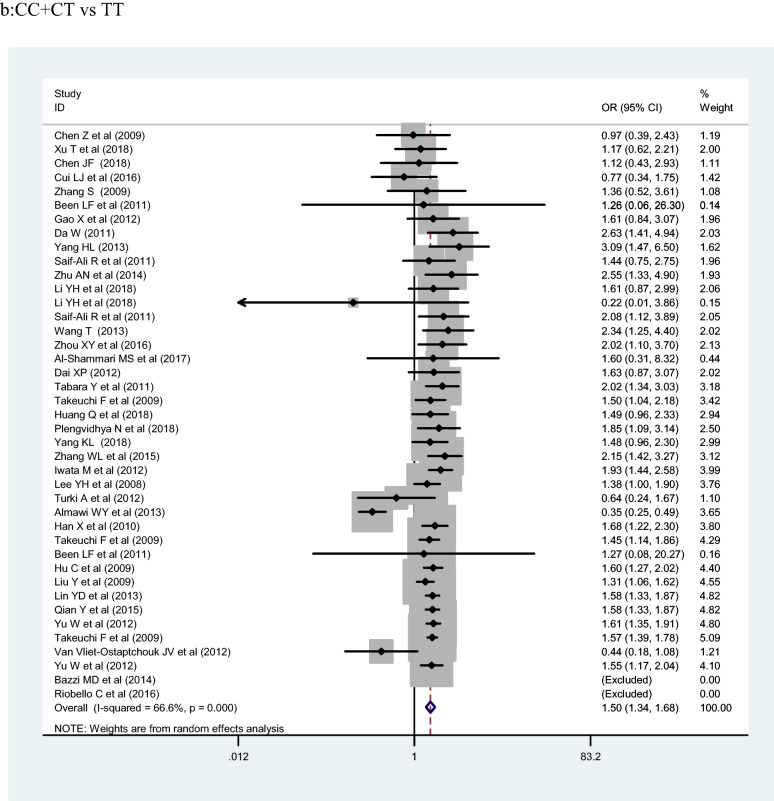

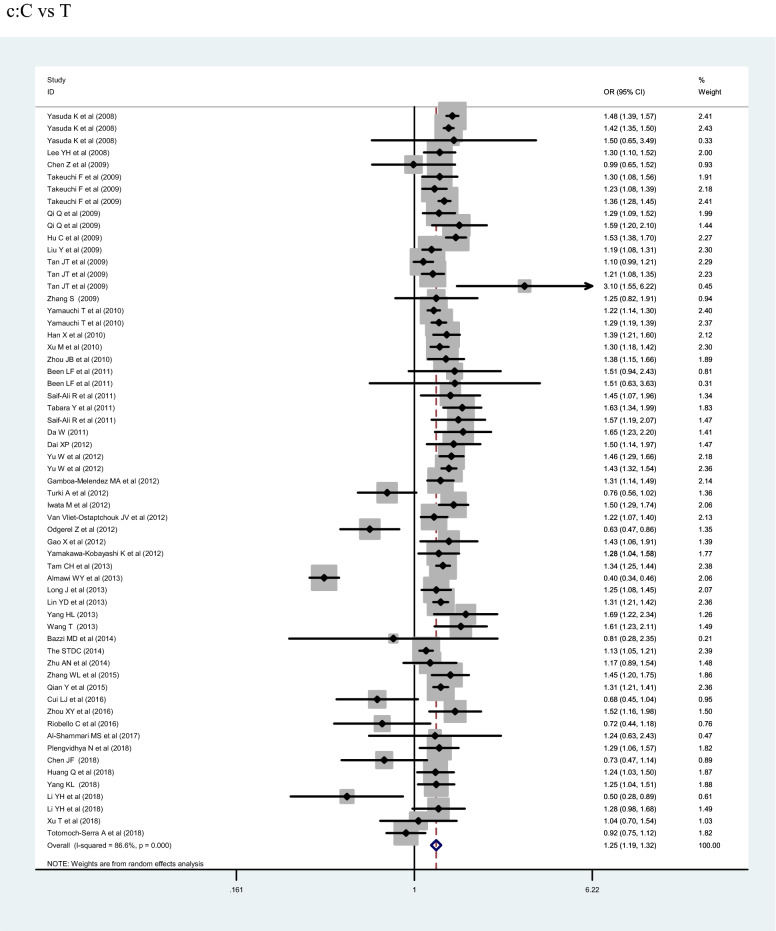
Forest plots of the KCNQ1 rs2237892 polymorphism under different genetic models.** a** is the model of CC vs CT + TT;** b** is the model of CC+CT vs TT;** c** is the model of C

The association of *KCNQ1* rs2237892 polymorphism with T2DM has been reported in many previous studies [21–62]. In 2008, two independently conducted genome-wide association studies (GWAS) in Japanese populations identified *KCNQ1* as a novel T2DM susceptibility gene [5, 6, 8]. Subsequently, the SNP locus rs2237892 of this gene was found to be correlated with the incidence of T2DM in Korean population [15]. In our present meta-analysis, there were 60 studies, 77,276 cases and 76,057 controls, that we evaluated for the possible association between *KCNQ1* rs2237892 polymorphism and T2DM risk. Our results showed that *KCNQ1* rs2237892 polymorphism could be associated with T2DM in the dominant (CC vs CT + TT), recessive (CC + CT vs TT) and allele models (C vs T). In a stratified analysis based on ethnicity, source of control, and case size, we found that *KCNQ1* rs2237892 polymorphism was significantly associated with T2DM in the dominant model, the allele model of East Asians, and in the allele model of West Asian populations. In Southeast Asian, South Asian, Caucasian, and other populations, *KCNQ1* rs2237892 polymorphism was not significantly related to T2DM. In the stratified analysis according to the source of control, we found that *KCNQ1* rs2237892 polymorphism was significantly correlated with T2DM in the dominant model and the allele model of HB and PB group, and in the allele model of NR group. But the correlation between *KCNQ1* rs2237892 polymorphism and T2DM in children lacked corresponding evidence. The stratified analysis of the sample size showed that the correlation between populations occurred when the number of samples in the case group was less than 500, within 1001–2000, and > 2000. The above analysis shows that the ethnicity, the source of the control group, and the sample size of the case group may be the factors in the association occurred (Fig. 2).

**Fig. 3:**
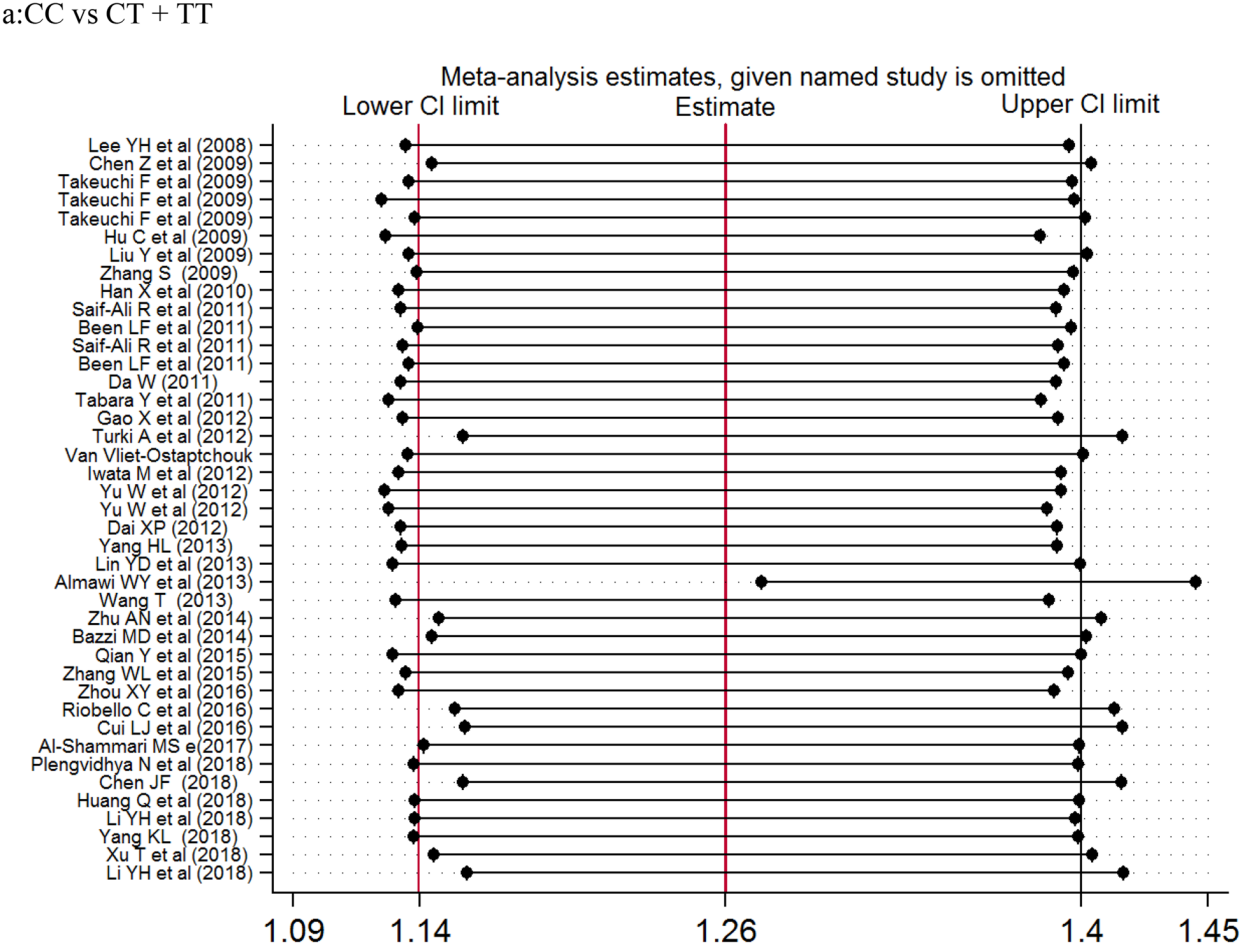

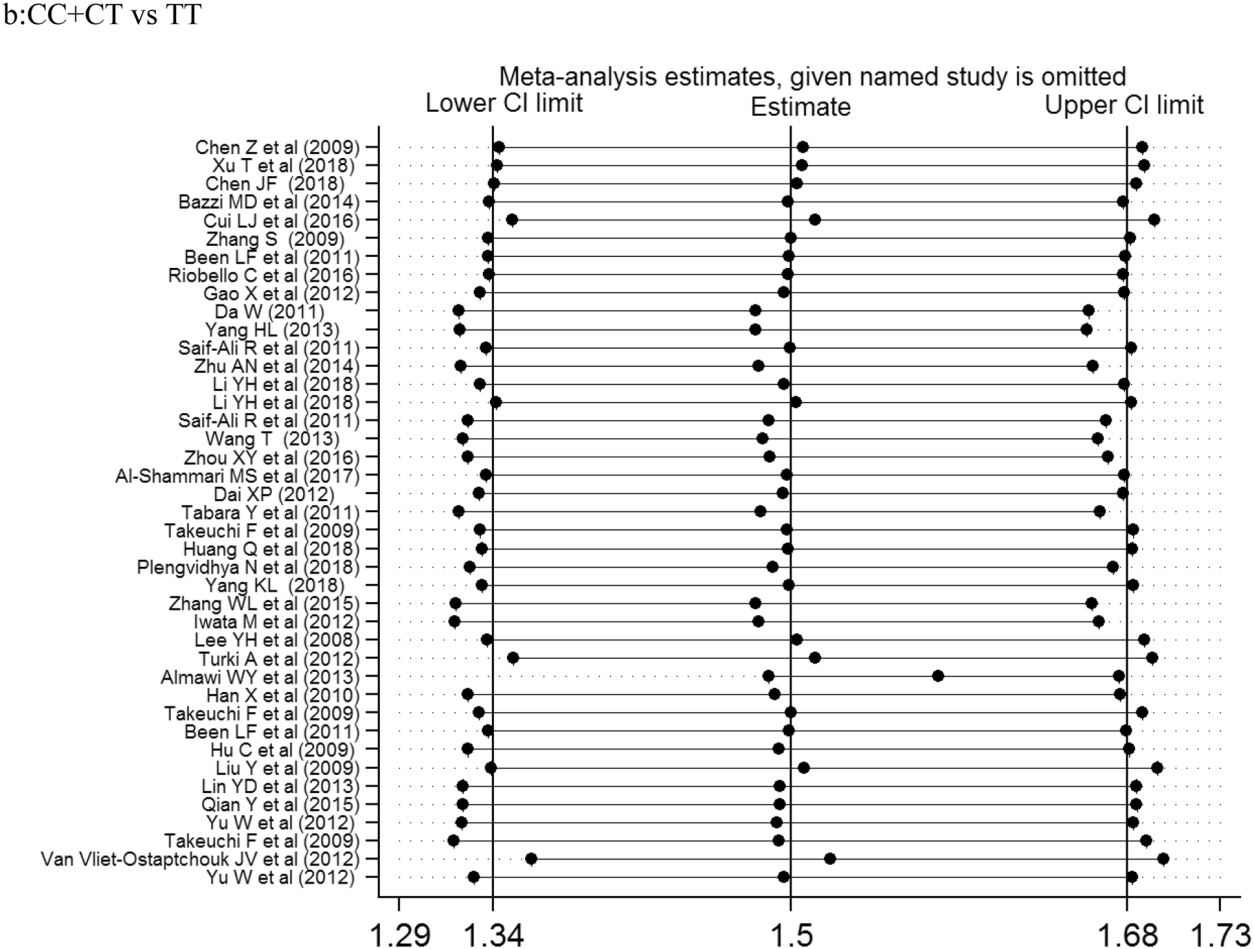

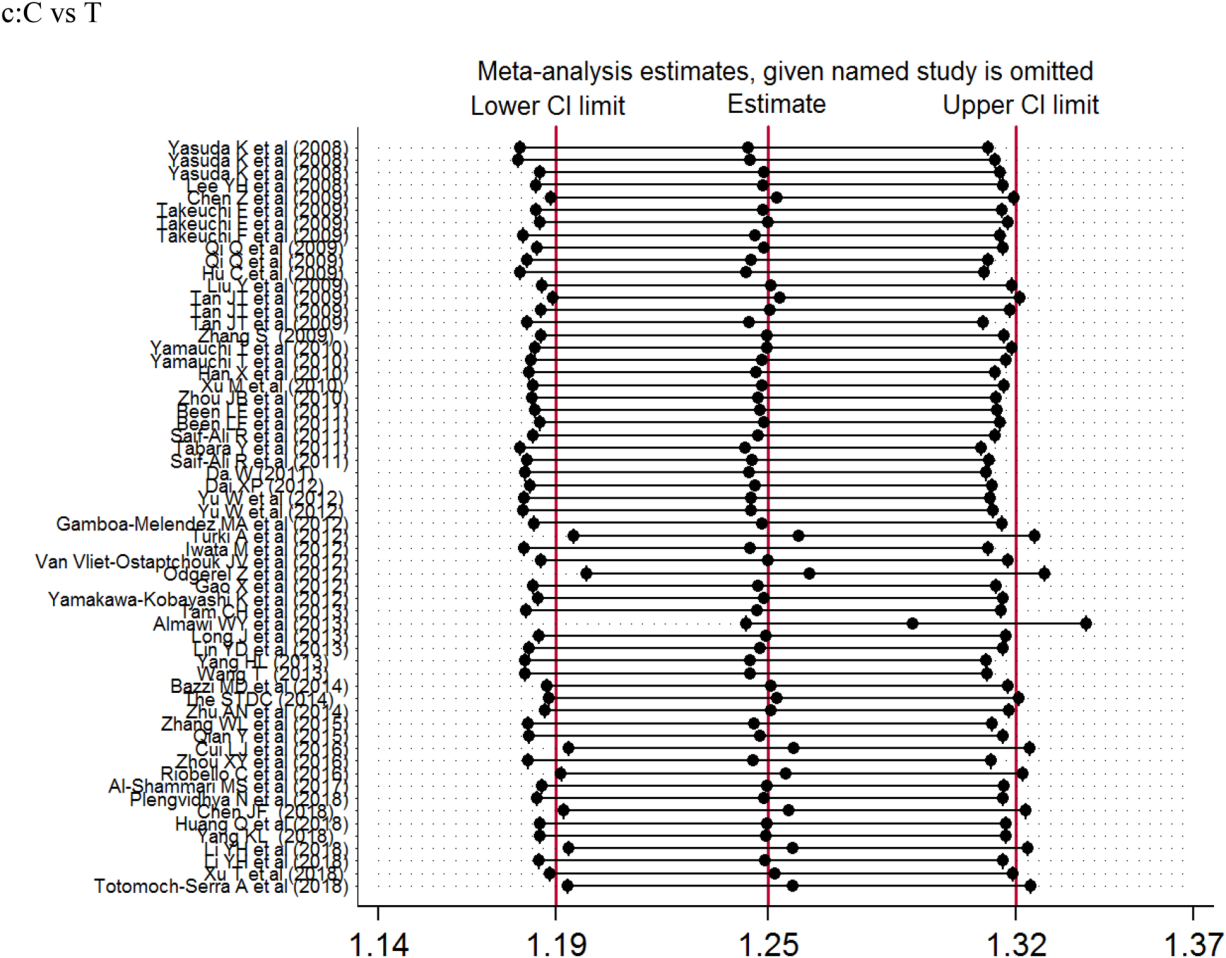
Sensitivity analysis examining the association between the KCNQ1 rs2237892 polymorphism and risk of stroke under these model.** a**CC vs CT + TT, ** b** CC+CT vs TT, ** c** C vs T

Previously, a meta-analysis was performed in 2012 to investigate the association between *KCNQ1* rs2237892 polymorphism and T2DM risk; however, only 25 articles were included in the analysis. Recently, a meta-analysis was performed to investigate the relationship between several *KCNQ1* SNPs and T2DM risk, and a significant relationship between *KCNQ1* polymorphism rs2237892 and T2DM risk was found [63]. However, the analysis was limited to 38 articles and incomplete sample size as well as selective bias are potential limitations of that study [63] (Fig. 3).

**Fig. 4:**
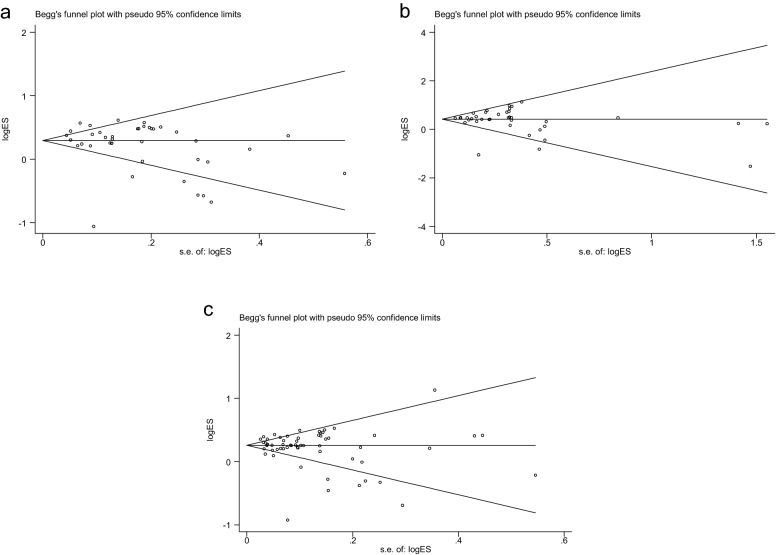
Begg’s funnel plot for publication bias analysis. ** a** is the model of CC vs CT + TT;** b** is the model of CC+CT vs TT;** c** is the model of C vs T

Compared to previous studies, our results demonstrate robust evidence to support a correlation between *KCNQ1* rs2237892 polymorphism and T2DM risk. Scientists do not currently understand the biological mechanisms that cause an association between *KCNQ1* and T2DM. There is biological evidence supporting the hypothesis that *KCNQ1* might play a role in the predisposition to T2DM. *KCNQ1*, encoding the alpha subunit of the IKsK + channel, is expressed in the tissues or cells of the heart [64], as well as in pancreas islets, which play an important role in the regulation of insulin secretion [23] (Fig. 4).

This meta-analysis has several limitations. Firstly, most of the articles included in the meta-analysis involved the Asian population, while there were few articles involving Caucasian and other populations. Therefore, we could not perform the analysis grouped by different populations, and the ability to apply our results to a more general population is subsequently limited. Secondly, T2DM is caused by complex interactions between genetic, lifestyle, and environmental factors. Our study focused exclusively on the impact of genetic factors on T2DM risk. In the future, further studies should be conducted to determine interconnection between *KCNQ1* rs2237892, lifestyle factors, and environmental factors on T2DM.

## Conclusion

Our meta-analysis demonstrated an association between *KCNQ1* rs2237892 polymorphism and the predisposition to T2DM. There was notable correlation between *KCNQ1* rs2237892 and T2DM in East Asian populations and West Asian populations. However, for the Southeast Asian, South Asian, Caucasian, and other populations, the relevance of the KCNQ1 rs2237892 SNP was not confirmed because of the relatively limited sample size and the sparse amount of research into this subject. In addition, the source of the control group and the sample size of the case would also have an impact on the study results in the stratified analysis of this study. Therefore, in future research, we suggest exploring the relationship between *KCNQ1* rs2237892 polymorphism and T2DM in a wide variety of populations. Although two meta-analyses were performed previously, the number of articles included in these was less than that in our study. Therefore, we believe that our study is superior than the two previous meta- studies.

## Data Availability

The datasets generated and/or analyzed during the current study are available from the corresponding author on reasonable request.

## Abbreviations

T2DM: Type 2 diabetes mellitus
IDF: International Diabetes Federation
PRISMA: Preferred Reporting Project
HWE: Hardy–Weinberg equilibrium
SNP: Single nucleotide polymorphism
